# Le zona ophtalmique: une dermatose exceptionnelle chez le nourrisson

**DOI:** 10.11604/pamj.2018.29.153.13216

**Published:** 2018-03-15

**Authors:** Bousayna Iraqi, Badr Sououd Benjelloun Dakhamaa

**Affiliations:** 1 Service des Urgences Médicales Pédiatriques Hôpital d’Enfants, Rabat, Maroc

**Keywords:** Zona ophtalmique, nourrisson, antiviraux, Ophthalmic zoster, infant, antiviral

## Abstract

We here report the case of a 9-month infant, born to a mother with a history of varicella in the third trimester of pregnancy but with no history of atopy, admitted to the emergency room with painful, pruritic rash in the right hemiface that had been ongoing for 4 days. During physical examination, the infant appeared to be in pain, with multiple cluster of grouped vesicles on erythematous skin in the right hemiforehead, in the right side of the nose and in the right cheek associated with edema of the upper and lower eyelids, with difficulty opening eyes and purulent conjunctival secretions. The infant was afebrile and in a good general condition. Ophthalmologic examination using the slit-lamp and fundus examination were normal. Complete blood count was normal. The diagnosis of ophthalmic zoster was retained on the basis of the clinical appearance of the lesions. The infant was treated with intravenous Aciclovir for 10 days associated with symptomatic local antiseptic treatment. Patient’s evolution was marked by the regression of vesicular lesions and of edema. Viral serologic test and rapid HIV test were negative. The particularity of our study is the occurrence of ophthalmic zoster in an immunocompetent infant, which is rare in children. We made three differential diagnoses which included Kaposi-Juliusberg syndrome, cutaneous infection due to herpes simplex virus and facial erysipelas.

## Image en médecine

Nous rapportons le cas d’un nourrisson âgé de 9 mois avec un antécédent de varicelle maternelle durant le troisième trimestre de grossesse, pas d’antécédent d’atopie personnelle, qui consulte aux urgences pour une éruption douloureuse et prurigineuse prenant l’hémiface droite évoluant depuis 4 jours. L’examen trouvait un enfant algique avec de multiples vésicules groupées en bouquet et reposant sur un fond érythémateux intéressant l’hémi front droit, le versant droit du nez, la joue droite avec présence d’un œdème des paupières supérieures et inférieures avec difficulté d’ouverture des yeux et sécrétions purulentes conjonctivales. Le nourrisson était apyrétique et en bon état général. L’examen ophtalmologique à la lampe à fente et au fond d’œil était sans particularité. Une numération formule sanguine a été réalisée et n’objectivait pas d’anomalies. Le diagnostic de zona ophtalmique a été retenu sur l’aspect clinique des lésions. Le nourrisson a été mis sous Aciclovir par voie intraveineuse pendant 10 jours en association avec un traitement symptomatique local à base d’antiseptique. L’évolution était marquée par la régression des lésions vésiculeuses et de l’œdème. Une sérologie virale et un test rapide VIH ont été réalisés et sont revenus négatifs. La particularité de notre observation est la survenue de zona chez un nourrisson immunocompétent, dans sa forme ophtalmique qui reste rare chez l’enfant. Les trois diagnostics différentiels étaient le syndrome de Kaposi-juliusberg, l’infection cutanée à herpès simplex virus et l’érysipèle de la face.

**Figure 1:**
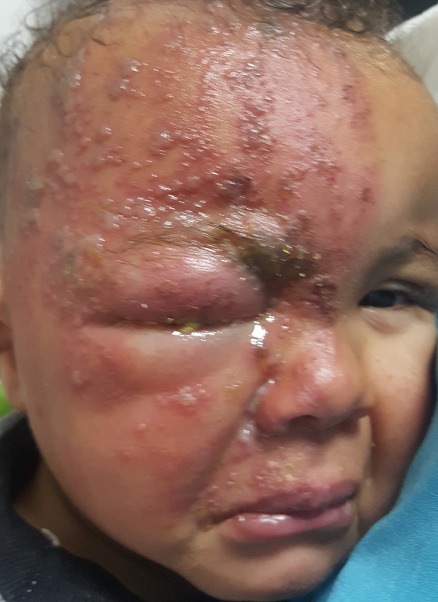
Multiples vésicules groupées en bouquet et reposant sur un fond érythémateux intéressant l’hémi front droit, le versant droit du nez, la joue droite avec présence d’un œdème des paupières supérieures et inférieures avec difficulté d’ouverture des yeux et sécrétions purulentes conjonctivales

